# Fabrication of phenyl boronic acid modified pH-responsive zinc oxide nanoparticles as targeted delivery of chrysin on human A549 cells

**DOI:** 10.1016/j.toxrep.2022.04.017

**Published:** 2022-04-22

**Authors:** Sushweta Mahalanobish, Mousumi Kundu, Sumit Ghosh, Joydeep Das, Parames C. Sil

**Affiliations:** a Division of Molecular Medicine, Bose Institute, P-1/12, CIT Scheme VII M, Kolkata 700054, India; b Department of Chemistry, Physical Sciences, Mizoram University, Aizawl 796004, Mizoram, India

**Keywords:** Chrysin, Lung cancer, Nanoparticle, Reactive oxygen species, Zinc oxide

## Abstract

Recently, different natural bioactive compounds have been used as anticancer agents for their various therapeutic benefits and non-toxic nature to other organs. However, they have various restrictions in preclinical and clinical studies due to their non-targeting nature and insufficient bioavailability. As a result, a zinc oxide nanoparticle (ZnO) based drug delivery medium was constructed which has good bio-compatibility and bio-degradability. It also displays cancer cell-specific drug delivery in a targeted and controlled way. In the present study, phenylboronic acid (PBA) tagged ZnO nanoparticles (ZnO-PBA) was fabricated and in the next step, chrysin (a natural bio-active molecule) was loaded to it to form the nanoconjugate (ZnO-PBA-Chry). Different characterization techniques were used to confirm the successful fabrication of ZnO-PBA-Chry. PBA-tagging to the nanoparticle helps in targeted delivery of chrysin in lung cancer cells (A549) as PBA binds with sialic acid receptors which are over-expressed on the surface of A549 cells. As ZnO dissociates in acidic pH, it shows stimuli-responsive release of chrysin in tumor microenvironment. Application of ZnO-PBA-Chry nanohybrid in lung cancer cell line A549 caused oxidative stress mediated intrinsic cell death and cell cycle arrest. ZnO-PBA-Chry downregulated MMP-2 and VE-Cadherin, thereby inhibiting metastasis and the invasive property of A549 cells.

## Highlights

• pH-responsive PBA functionalized ZnO nanoparticle was fabricated.
• Chrysin was loaded as a bioactive anticancer agent into ZnO nanoparticle.
• ZnO-PBA-Chry induced intrinsic cell death and cell cycle arrest in A549 cells.
• It inhibited metastasis and invasive properties of A549 cells.

## Graphical Abstract

**Figure:**
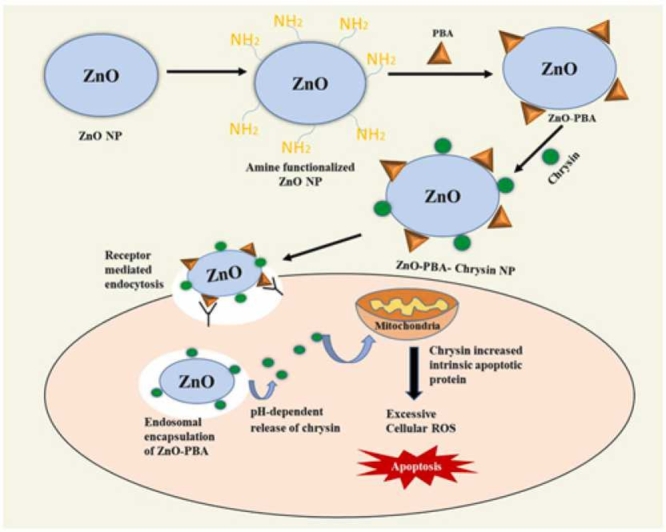

## Introduction

Lung cancer is a serious type of cancer due to its association with a high rate of global morbidity. Especially, non-small-cell-lung cancer contributes to 85% of the lung cancer cases where various chemotherapy and radiation therapy are extensively used for treatment purpose. Chemotherapeutic agents exert the most promising modality to induce cytotoxicity in neoplastic cells by targeting specific receptors, DNA or proteins of these cancerous tissues. However, the available pharmaceutical agents for chemotherapy exhibit non-targeted effects and have a wide range of cytotoxicity in various organs [1], [2], [3]. Moreover, the application of these drugs at higher doses in tumor site is restricted due to the high drug efflux ability and development of drug resistance by cancer cells [4]. Various members of the multidrug resistance-associated proteins (MRPs) utilize ATP to eliminate a wide range of chemotherapeutic agents across the cell membranes [5], [6], [7]. Consequently, to overcome these limitations, extensive research works have been carried out for the development of a unique therapeutic regimen that can exert cytotoxicity only upon cancer cells without effecting normal tissue homeostasis. Some natural products have gained attention as therapeutic agents as they have multi-modal properties and minimal toxic effects [8]. Among them, flavonoids have been extensively studied as a chemotherapeutic agents against a wide array of cancers [9]. Chrysin (5, 7-dihydroxyflavone), a secondary metabolite of the flavone group, presents in blue passion flower (*Passiflora caerulea*), mushroom, honey, propolis, etc. Previous studies have demonstrated that chrysin possesses anti-inflammatory, anti-oxidant, anti-proliferative, and cytotoxic anti-tumor properties [10], [11], [12]. Two major metabolites of chrysin are chrysin glucuronide and chrysin sulfate, which are substrates for multidrug resistance-associated protein 2 (MRP2) [13]. MRP2, as a glutathione transporter, exports GSH in the transport process. Therefore, chrysin metabolites cause a significant level of intracellular GSH efflux from the cells and resulting intracellular GSH depletion and overproduction of reactive oxygen species (ROS) in cancer cells [14]. But the poor bioavailability of chrysin due to extensive metabolism limits its application in cancer treatment [15]. To resolve this issue, the nanoparticle mediated drug delivery system has been used to reduce these limitations by increasing the bioavailability. Chrysin has also been stated as an effective anticancer drug candidate for delivery through nanocarriers [16].

Among various nanoparticles, inorganic metal oxides are widely used as they are small in size and also have favorable surface chemistry. It has been reported that zinc oxide nanoparticles (ZnO NPs) exhibit higher selectivity, retention, and controlled drug release properties [17], [18]. In human body, zinc is an indispensable trace element required for the activation of various enzymes like carbonic anhydrase, carboxy peptidase, and alcohol dehydrogenase. High biocompatibility and reduced side effect of ZnO NPs is reported [19]. The rapid dissolution of ZnO NPs to Zn^2+^ ions take place in acidic pH and this nature makes it suitable for use as nanocarriers in acidic tumor microenvironment. ZnO NPs can induce cytotoxicity to tumor cells by upsurging ROS production, DNA damage, and finally apoptosis mediated cell death [20]. Additionally, ZnO has high biocompatibility, easy to synthesize and it can also be identified easily as it has intrinsic fluorescence property. All these properties make ZnO NP a suitable nanocarrier in cancer therapy.

Tumor cells exhibit enhanced permeability and retention (EPR) effect i.e., which allows selective extravasation and retention of macromolecular drugs. This EPR property allows the extravasation and accumulation of NPs in tumor site by passive route. But, on the basis of pathophysiological characteristics (like pH, temperature, etc.), passive targeting of NPs to tumor site has several drawbacks. The lack of specificity impedes the delivery of NPs to the tumor tissue. Moreover, the high density of cells in tumor and increased interstitial fluid pressure hinder the proper penetration and homogeneous distribution of NPs inside the tumor [21]. To overcome this situation, some molecules specific to the receptor overexpressed in tumor tissues are linked with the nanocarriers [22]. Among these ligands, 3-carboxybenzeneboronicacid (PBA) is used as a targeting molecule in tumor tissues [23].

During malignant transformation, glycosylation is heavily altered, that leads to upregulation of sialylated glycans on cancer cell surface [24]. The interaction of exocyclic polyol group of sialic acid (SA) and the boronic acid group of PBA via reversible cyclic boronate ester bonds facilitate PBA mediated tumor targeting [25]. Although PBA interacts with other common sugars, stable complexes form only at pH greater than the pKa value of PBA during the interaction. On the other hand, PBA forms a stable complex with SA also at pH less than its pKa value [26]. This particular feature of PBA offers the molecular basis of certain SA detection at biological pH. PBA has not only this specificity and high binding ability with SA, it has also several advantages as it is of low cost, non-immunogenic, and non-toxic.

Herein, we developed PBA functionalized ZnO NPs which were further loaded by chrysin. PBA moiety acts as a targeted molecule as SA is overexpressed on the surface of A549 cells. ZnO nanocarriers mediated targeted delivery enhances the cellular uptake of chrysin in vitro and diminishes cell viability.

## Material and methods

### Materials

Zinc(II) acetate dihydrate (99.5%), magnesium(II) acetatetetrahydrate (99%), 3-carboxybenzeneboronic acid (97%), sodium chloride, *N*-hydroxyl succinimide (NHS), 1-(3-Dimethylaminopropyl)-3-Ethyl Carbodiimide hydrochloride (EDC.HCl) (99%) potassium hydroxide (KOH) and 3-(4,5-Dimethylthiazol-2-yl)-2,5-diphenyltetrazolium bromide (MTT) were bought from SRL (Mumbai, India). Chrysin and (3-Aminopropyl) triethoxysilane (APTES) (99%) were acquired from Sigma-Aldrich (St. Louis, Missouri, United States). Ethyl alcohol (ETOH) and dimethyl sulphoxide (DMSO) were acquired from Merck (Kenilworth, New Jersey, United States). RPMI-1640, MEM, trypsin and antibiotics were acquired from HIMEDIA (Mumbai, India).

### Methods

#### Preparation of amine-conjugated ZnO NPs

ZnO and amine-conjugated ZnO NPs (NH_2_-ZnO) synthesis were accomplished by monitoring the protocols as described elsewhere [27], [28], [29]. Initially, zinc acetate (2.0 mmol) and magnesium acetate (44 mg) were mixed in anhydrous ethanol (50 mL). Then the reaction mixture was refluxed at 60 °C. After 5 h, ethanolic KOH was added drop by drop, and the reaction was continued for 2 h in stirring condition to achieve ZnO NPs. In the final stage, 3-aminopropyltriethoxysilane (APTES) (1 mL) was added to the solution in stirring condition at room temperature and the reaction was continued for 1 h to obtain NH_2_-ZnO NPs.

#### Tagging of PBA to NH2-ZnO NPs (ZnO-PBA)

The formulation of PBA tagged nanocarrier was achieved following our previous literature [28]. Initially, PBA was activated by adding EDC (40 mg) and equivalent NHS in DMSO and the reaction mixture was sustained in stirring condition. After 3 h, amine tagged ZnO NPs solution was mixed to the previous mixture. After 24 h, ZnO-PBA was obtained as a final product.

#### Loading of chrysin to formulate ZnO-PBA-Chry

To load chrysin to the ZnO-PBA, chrysin was dispersed in DMSO. After that, the dispersed solution of chrysin was added to ZnO-PBA. The reaction was kept in stirring condition which was continued for 24 h. The amount of chrysin-loading was measured at 290 nm with UV–VIS spectroscopic technique. The drug loading content (DCC) and drug entrapment efficiency (DEE) were assessed following the equation:

DLC (%) = [(weight of drug in nanoparticles) / (weight of nanoparticles taken)] × 100

DEE (%) = [(weight of drug in nanoparticles) / (weight of drug injected)] × 100

#### Cell culture

The human adenocarcinomic alveolar basal epithelial cell (A549) and human lung epithelial cell line (L132) were cultured in DMEM containing 10% FBS and 100,000 U/L of penicillin and 100 mg/L of streptomycin within an incubator (37 °C) enriched with 5% CO_2_. Confluent monolayers of A549 and L132 cells (60%−80%) were subjected to different treatments.

#### Cell viability assay

A549 and L132 cell viability were determined by using MTT assay following the manufacturer's protocol. Briefly, 96-well plates were used to seed the cells and when 70% confluence was reached, different concentrations of treatment regimens were used for 48 h. Then, MTT solution (0.5 mg/mL) was used to incubate the cells for 4 h and then DMSO solution was added to dissolve the formazan crystals. Then, intensity was observed on a microplate reader at 570 nm.

#### Cellular uptake of NPs in vitro

A549 cells were cultured within 6 well plates. Then, they were treated with ZnO nanoparticles and incubated for 48 h. After this, the media was discarded. Finally, BD FACS Calibur system was used to perform the flow cytometry under the FITC filter.

#### Cell cycle arrest

After the nanoparticle treatment period was over, PBS wash was done for A549 cells and trypsinized and fixed with 100% methanol for 10 min at − 20 °C. Following PBS wash, 100 µg/mL RNase A was used to treat the cells. Then, PI (0.05 mg/mL) was used for staining the DNA. After 30 min incubation in PI at 37 °C, flow cytometry was performed and cellular percentage in each phase of the cell cycle was determined.

#### Detection of intracellular ROS and mitochondrial membrane potential (MMP)

To find out nanoparticle mediated oxidative insult in A549 cells, ROS and MMP assay were performed via FACS method. The protocol for FACS analysis was described elsewhere [30]. A549 cells were subjected for culture in 60 mm culture dish. When the cells reached at 80% confluency, ZnO, free chrysin, and ZnO-Chry were used for cellular treatment. The cells were treated with IC50 dose of chrysin, i.e., 68.28 μg/mL for free chrysin, 155.14 μg/mL for ZnO, 223.42 μg/mL for ZnO-PBA-Chry (calculated from DLC value). The equivalent concentration of chrysin was used in case of ZnO and ZnO-PBA- Chry treated group. After 48 h treatment period, the cells were stained with DCFDA and JC-1, respectively and kept in 30 min incubation at room temperature in the dark. Finally, BD FACS Calibur system was used to perform flow cytometry under the FITC filter.

#### Wound healing assay

Bidirectional wound healing assay was used to investigate the migration of A549 under different conditions. Briefly, after reaching 80–90% confluence, monolayer of A549 cells was subjected for scratching via a sterile pipette tip to create a bidirectional wound. In control well, migration rate was considered as 100%, and based on this, the healing frequency to other plates was determined [31].

#### Detection of cellular apoptosis

After treatment protocol, A549 cells were collected by centrifugation and to the resulting precipitate, 500 µL of 1X binding buffer was added. 5 µL of annexin V-FITC and 5 µL of propidium iodide were mixed and the suspension was incubated for 10 min at room temperature in the dark [32].

#### Western blot

To perform western blot analysis, equivalent concentration of lysate was loaded and resolved in SDS-PAGE (10–12% as required). Then the protein was transferred into PVDF membrane and subjected for blocking (2% BSA) in room temperature for 1 h. Then, the membrane was incubated with primary antibody dilution buffer at 4 °C for overnight. Next day, after TBST washing, incubation of the membrane with HRP conjugated secondary antibody was done for 2 h at 37 °C. Then after TBST wash, the membrane was developed using ECL solution.

#### Statistical analysis

Three independent experiments were performed and the results were expressed as their mean data±SD. To evaluate the data statistically, one-way analysis of variance (ANOVA) and Tukey test were performed. p-value ≤ 0.05 was considered as statistically significant.

## Results and discussion

### Formulation and characterization of ZnO, ZnO-PBA, and ZnO-PBA-Chry NPs

In the current study, amine functionalized ZnO NPs were prepared in the first step following the protocol of our previous manuscript [27], [33]. To achieve targeted drug delivery, PBA-ZnO was formulated through the conjugation of PBA to the amine functionalized ZnO NP. In the final step, chrysin was conjugated to the ZnO-PBA via formation of covalent bond with the metal ion.

**Fig. 1:**
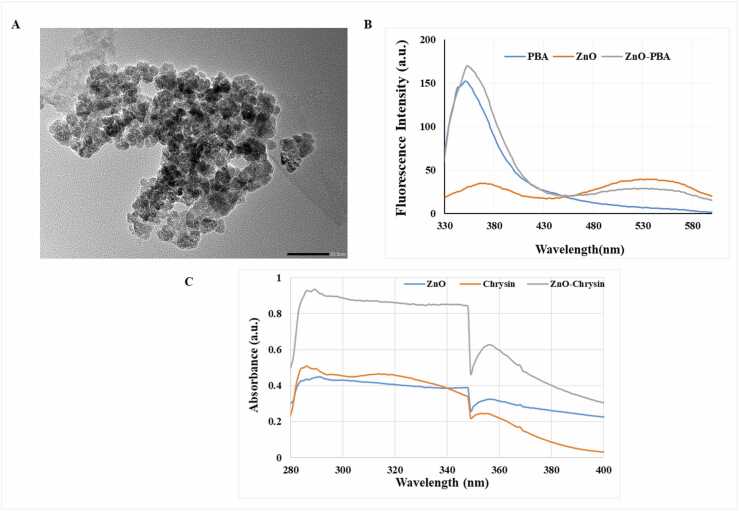
(A) TEM of ZnO-PBA-Chry NPs. (B) Fluorescence spectra of ZnO (orange), PBA (blue) and ZnO-PBA NPs (gray). (C) UV–VIS spectra of ZnO (blue), Chrysin (orange) and ZnO-Chry (gray).

The calculated DLC and DEE were noticed to be 30.56% and 44% (Table1), respectively. The size of the nanoparticle was measured by using Transmission electron microscope (TEM; Zeiss-EM10C-100 KV, Japan). The average particle diameter was 25–30 nm (Fig. 1A).

**Table 1:** Drug loading content (DLC) and Drug entrapment efficiency (DEE) of ZnO/Chrysin nanoparticles.

Feed Ratio, ZnO: Chrysin	DLC%	DEE%
1:1	30.56	44.00

UV–VIS spectroscopic experiment and fluorescence experiment were executed to study the optical properties of the newly synthesized nanoparticles. Absorbance of the synthesized nanoparticles was measured through UV–VIS spectroscopy (Shimadzu spectrophotometer). The nature of fluorescence spectrum was presented in Fig. 1B. PBA displayed one sharp emission peak at ~ 345 nm when it was exposed at 310 nm [34]. In contrast, ZnO flashed two emission peaks at ~ 345 nm and ~ 530 nm under the same excitation. The first peak intensity (i.e., at ~ 345 nm) of ZnO-PBA was higher compared to the second peak which was observed at ~530 nm. The intensity of the first peak was amplified due to the existence of PBA in ZnO-PBA. The result suggested that the conjugation of PBA with the nanohybrid was successfully synthesized (Fig. 1C). ZnO gave a characteristic absorption peak at ~348 nm and it was almost similar to the previous report [35].

**Fig. 2:**
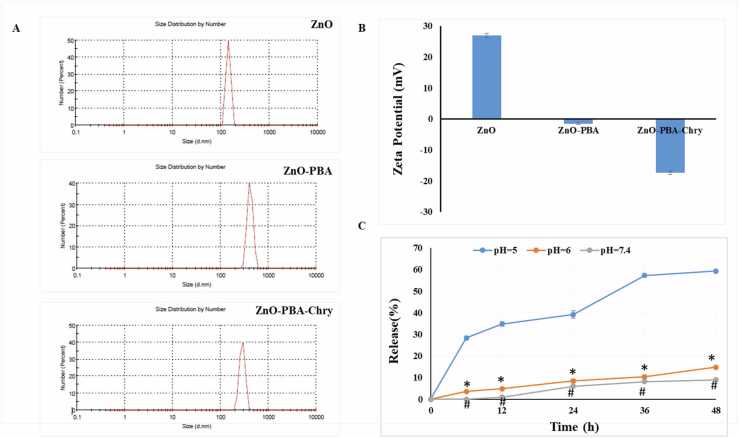
(A) Dynamic Light Scattering and (B) Zeta Potential of ZnO, ZnO-PBA and ZnO-PBA-Chry. (C) Time-dependent release of Chrysin from the nanoconjugate at various pH: 5 (blue), 6 (orange) and 7.4 (gray). All values are expressed as mean ± SD. “*” and “#” represents significant differences with respect to pH = 6 and pH = 7.4 respectively.

The hydrodynamic diameter and surface charge of the NPs were calculated by dynamic light scattering (DLS) instrument (Delsa™ Nano C particle size analyzer, Beckman Coulter, Brea, CA, USA). The result was highlighted in Fig. 2A. The measured hydrodynamic sizes were 142.5 nm, 285.1 nm, and 415.1 nm for ZnO, ZnO-PBA, and ZnO-PBA-Chry respectively. The result indicated step by step incorporation of PBA and Chrysin to the ZnO NPs, respectively. At the time of measurement of hydrodynamic size, extensive hydration of nanoparticle had taken place and due to that reason, the measured particle size in DLS appeared to be larger than that measured by TEM analysis [36].

The stepwise incorporation of PBA and chrysin was further confirmed by the measurement of hydrodynamic diameters through dynamic light scattering (DLS) experiment. The particle diameters increased after the step by step attachment of PBA and chrysin to the ZnO NP. The size of NP figured higher under DLS than that obtained from TEM analysis. This happened due to the presence of solvation/hydration of the nanoparticles in DLS measurement. The result obtained from DLS experiment was presented in Fig. 2A. Zeta potential result was presented in Fig. 2B. The zeta potential study was performed in neutral pH condition. The zeta potential of ZnO, ZnO-PBA, and ZnO-PBA-Chry were + 26.86, − 1.53, and − 17.36 mV, respectively. The result indicated the stepwise grafting of PBA and chrysin. Initially, due to the presence of a positively charged amine group in the surface of ZnO NPs, zeta potential showed value in the positive side. On the other hand, the value was switched from positive direction to negative direction after the conjugation of PBA, as it has negatively charged carboxylic group. The value was further shifted in the negative direction after the successful grafting of chrysin to the surface of nanoparticles, as chrysin consists of ionizable hydroxyl group. The stability of the nanoformulations was also examined in a solution and the solution mimics in vivo system. Initially, the nanoconjugate was suspended in water which contained 10% FBS. Then, the changing of hydrodynamic diameter and PDI value with time was evaluated. The diameter of ZnO-PBA-Chry was noticed to be 428.56 ± 37.06 nm, 430.63 ± 33.20 nm, and 436.46 ± 43.88 nm and the average PDI values were observed to be 0.674, 0.691 and, 0.672 at day 0, day 7 and day 14, respectively. The result was displayed in Table 2. The hydrodynamic size remained almost constant with time, thereby signifying good stability of the nanoformulations in solution. Additionally, no significant change was detected in the PDI values of the nanohybrid in solution after 7 and 14 days, designating further stability of ZnO-PBA-Chry.

**Table 2:** Time dependent change of hydrodynamic size and PDI of the nanoconjugates.

	Day 0		Day 7		Day 14	
ZnO-PBA-Chry	Size	PDI	Size	PDI	Size	PDI
**Average**	428.56	0.674	430.63	0.691	436.46	0.672
**SD**	37.06	0.1289	33.20	0.1450	43.88	0.0420

### pH-dependent chrysin release study with time

Chrysin loaded ZnO-PBA NPs was designed to form a chelate complex with Zn^2+^ ion [51,52]. In alkaline solution, the Zn^2+^-chrysin complex remains stable as the hydroxyl groups (-OH) of chrysin stay mainly in ionized form (O^-^), as a result, it behaves as a suitable ligand for the formation of chelate complex. Chrysin was released from the nanoconjugate when the complex was broken. In acidic pH, Zn^2+^-Chrysin complex is unstable as chrysin mainly acts as a poor ligand due to the existence of its unionized form. ZnO NPs partially dissolute in this form. In dialysis bag, nanoconjugates containing 1 mg/mL chrysin was suspended in 10 mL buffer solution with continuous shaking at 100 rpm. The amount of chrysin which was released in different pH values with time was analysed using UV–VIS spectrophotometric instrument. The result was presented in Fig. 2C. Release amount of chrysin was noticed to be ~ 28% at pH 5.0 after 6 h, while very low amount of chrysin release appeared at pH 7.4. To fulfill the experiment, three solutions with different pH values (endosome pH 5.0, physiological pH 7.4 and intermediate pH 6.0) were considered. The amount of chrysin release was found to be almost 59% after 48 h at pH 5.0. On the other hand, it was noticed to be almost 14% and 9% at pH 6.0 and pH 7.4, respectively after the end of 48 h. As a result, the nanoconjugate could be efficiently applied as a pH-dependent drug delivery system.

### Cellular uptake study

**Fig. 3:**
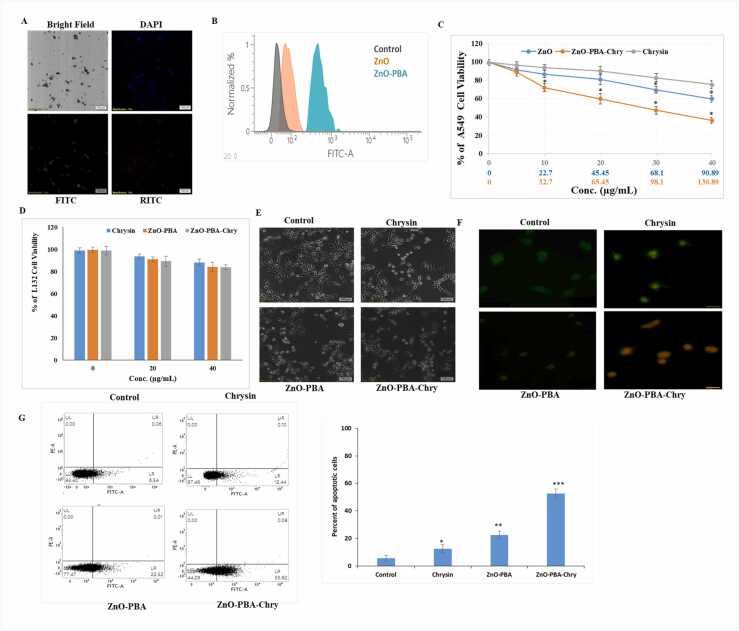
(A) Fluorescence microscopic image of PBA-ZnO-Chry. NPs obtained at 40× magnification (B) Intracellular uptake of ZnO and ZnO-PBA in A549 cells (C) MTT assay to detect viability of A549 cells. All values are represented as mean ± SD. “*” represents significant differences with respect to ZnO (D) MTT assay to detect viability of L132 cells. (E) Pictorial micrographs of A549 cells obtained at 10× magnification. (F) Acridine orange ethidium bromide staining in A549 cells (G) Percentage of cellular apoptosis by FACS analysis. All values are expressed as mean ± SD. “*” values differ significantly from control *(p ≤ 0.05), ** (p ≤ 0.01), *** (p ≤ 0.001).

Under fluorescent microscope (40×), the synthesized nanohybrids emitted a blue (DAPI) and green (FITC) fluorescence (Fig. 3A) which indicated its implication as a bio-imaging agent. ZnO and ZnO-PBA NP’s intracellular uptake in A549 cells was checked via FACS analysis (Fig. 3B). The fluorescence intensity indicated nanoparticle internalization within the cells. The increased green fluorescence intensity indicated the greater uptake efficacy of ZnO-PBA inside A549 cells.

Various studies reported the increased expression of SA in the plasma membrane of cancer cells in comparison to the normal one [37], [38]. This SA overexpression accelerated the proliferation and metastasis of cancer cells. PBA can specially interact with the membrane bound SA. This feature of PBA rendered the higher intracellular uptake of PBA tagged nanoparticles by the cancer cell in comparison to untagged nanoparticles [39].

### Dose dependent cytotoxicity of ZnO-PBA-Chry nanohybrids

The cytotoxicity of the nanoconjugates on A549 and L132 cell line was carried out by MTT assay. ZnO-PBA-Chry exhibited dose dependent cytotoxicity from 16.3 to 130.8 μg/mL dose which is equivalent to 5–40 μg/mL of free chrysin. Treatment of ZnO-PBA-Chry on A549 cells indicated a combinatorial effect of ZnO-Chry and free chrysin (Fig. 3C). However, free chrysin, ZnO-PBA, ZnO-PBA-Chry did not exhibit significant level of cytotoxicity in normal alveolar epithelial cell line L132 (Fig. 3D). The morphological alteration of A549 cell was observed under bright field microscopy. The cytotoxic effect of ZnO-PBA-Chry was found to be higher in comparison to ZnO-PBA and free chrysin treated cells (Fig. 3E). Nanohybrid mediated cytotoxicity causes the cellular apoptosis by inducing cellular shrinkage, membrane blebbing, etc. [40]. AO/EtBr staining in A549 cells followed by 24 h incubation with free chrysin, ZnO-PBA, and ZnO-PBA-Chry showed a gradual increase of apoptotic characteristics (Fig. 3F). Early stage of apoptotic cells were marked by yellow-green acridine orange stain due to chrysin and ZnO-PBA treatment where late stage apoptotic cells showed orange stain after ZnO-PBA-Chry treatment. Annexin V-FITC apoptotic assay showed an increased percentage of apoptotic cells due to translocation of phosphatidyl serine from the inner leaflet to the outer leaflet of the membrane. The percentage of apoptotic cells increased significantly from 6.54% in the control to 12.44%, 22.52%, and 55.62% after free chrysin, ZnO-PBA, and ZnO- PBA-Chry treatment (3G).

The essential criterion to become an anti-cancer drug is the cytotoxic potential upon cancerous cells. ZnO-PBA-Chry nanoparticles effectively induced cytotoxicity in A549 cells. Therefore, considering the anti-cancer efficacy of chrysin and ZnO nanoparticles, we further investigated the molecular mechanisms of the cytotoxic effects.

### ZnO-Chry nanohybrid caused oxidative stress mediated intrinsic cell death and cell cycle arrest

**Fig. 4:**
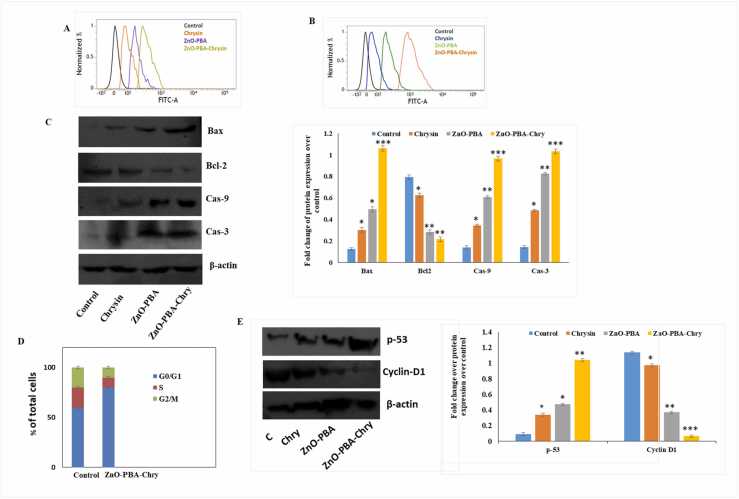
(A) Intracellular ROS measurement by DCFDA assay (B) Mitochondrial membrane potential using JC-1 dye by FACS analysis (C) Immunoblot and densitometric analysis of intrinsic apoptotic proteins Bax, Bcl-2, Caspase-9, Caspase-3. (D) Cell cycle progression analysis (E) Immunoblot and densitometric analysis of p-53, Cyclin-D1. β-actin was used as a loading control. All values are expressed as mean ± SD. “*” values differ significantly from control *(p ≤ 0.05), ** (p ≤ 0.01), *** (p ≤ 0.001).

To investigate the cause of cytotoxicity of ZnO-PBA-Chry in A549 cells, intracellular ROS and MMP were measured by using DCFDA and JC-1 dye respectively. Consistent with our MTT cell survival result, it was observed that ZnO-PBA-Chry upregulated intracellular ROS production dose dependently (indicated by increased green fluorescence intensity) (Fig. 4A). The decreased MMP in ZnO-PBA-Chry treated A549 cells indicated nanohybrid mediated mitochondrial dysfunctions (Fig. 4B). After ensuring the altered mitochondrial health by MMP assay, we performed immunoblot analysis of several proteins associated with intrinsic cell death machinery. The Bcl-2 downregulation and Bax, Caspase 9 and Caspase 3 upregulation in ZnO-PBA-Chry treated A549 cells was observed (Fig. 4C). Due to the combinatorial effect of ZnO-PBA-Chry, it was more potent to induce ROS, MMP, and intrinsic cell death machinery compared to ZnO-PBA or free chrysin in A549 cells.

The upregulation of ROS inside the cells triggers the onset of oxidative stress [41]. ROS production at basal level can induce cellular differentiation. But exaggerated ROS generation beyond threshold limit causes irreversible damage in tissue, resulting in cell death [8]. Oxidative stress is responsible for the downregulation of mitochondrial membrane protein- Bcl-2 and the upregulation of Bax. The disrupted Bax/Bcl-2 ratio along with Cytochrome C released from mitochondria to cytosol induces the activation of Caspase 9 and 3 to trigger intrinsic mode of apoptosis [42].

Cancer cells exhibit abnormal properties of cellular proliferation. Anti-cancerous agents can arrest the progression of cell cycle at S, G2, or M phase. Flow cytometric analysis showed that nanohybrid exerts inhibitory effects on A549 cell cycle progression. It has been found that chrysin nanohybrid effectively suppressed cell cycle progression of A549 cells in G0/G1 phase (Fig. 4D). Arrest of cell cycle in G0/G1 phase is due to the accumulation of p53 which in turn downregulated the expression of cell regulatory molecule, Cyclin D1. Immunoblot analysis confirmed upregulated expression of p53 and downregulated expression of Cyclin D1 which is responsible for cell cycle arrest in G0/G1 phase (Fig. 4E).

The imbalance between oncogenes and tumor suppressing gene activity is the hallmark of cancer progression [43], [44]. p53, a tumor suppressor gene, is important for cellular viability. Activation of p53 is responsible for triggering apoptosis. In addition, p53 also modulates the activity of various molecules associated with mitochondria-dependent apoptotic pathways [45], [46], [47]. Following nanohybrid exposure, p53 upregulation caused cyclin D1 inhibition and cell cycle arrest.

### ZnO-PBA-Chry mediated cell migration retardation

**Fig. 5:**
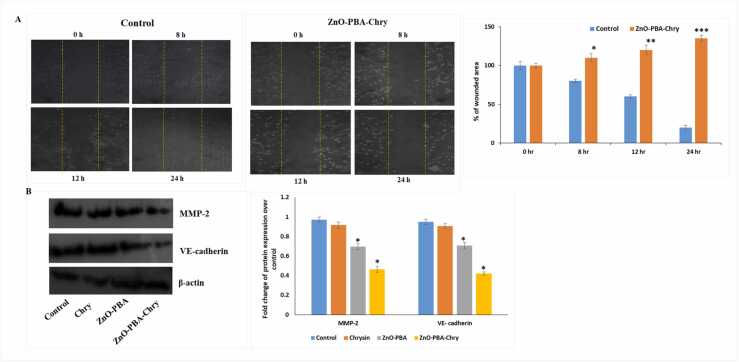
(A) Effect of ZnO-PBA-Chryin on cellular migration under phase contrast microscopy and the percentage of wounded area (B) Immunoblot and densitometric analysis of MMP-2 and VE-cadherin. β-actin was used as a loading control. All values are expressed as mean ± SD. “*” values differ significantly from control *(p ≤ 0.05), ** (p ≤ 0.01), *** (p ≤ 0.001).

To find out the altered cellular motility of A549 cells due to proliferative changes, wound healing assay was performed. Untreated A549 cells gradually migrated and filled up the wounded region (Fig. 5A). But, after nanohybid treatment (well below its LC50 value), A549 was unable to grow in the wounded region and there was a significant decrease of cellular density. Therefore, these results indicated ZnO-PBA-Chry can induce oncogenic properties to A549 cells.

After inspecting ZnO-PBA-Chry mediated anti-migratory effect in A549 cells via wound healing assay, we further intended to study the immunoblot analysis of various proteins associated with cellular invasion and migration. Immunoblot analysis showed that ZnO- PBA-Chry successfully reduced the upregulated MMP-2 expression of A549 cells. Moreover, the expression of epithelial to mesenchymal marker VE-cadherin reduced dose dependently after the treatment of chrysin, ZnO-PBA, and ZnO-PBA-Chry (Fig. 5B).

Invasion and metastasis are two characteristic features of cancer cells. MMP-2 and MMP-9 are two important enzymes that help to invade the tumors [48]. The invasive ability of tumor cells is closely associated with vasculogenic mimicry, an alternative process of blood vessel formation by aggressive tumor cells for their survival, growth, and metastasis. Vascular-associated gene such as VE-cadherin is responsible for vasculogenic mimicry [49], [50]. ZnO-PBA-Chry could effectively retard the cell migration by downregulating MMP-2 expression and inhibited the cellular invasion by reducing VE-cadherin expression.

## Conclusion

From our study, it has been found that synthesized ZnO NPs can be used to inhibit A549 cell survival. PBA conjugation helps to increase drug absorption efficacy in A549 cells as PBA interacts with SA which is overexpressed in A549 cell surface. ZnO based nanoformulation considerably increased the anticancer efficacy of chrysin by enhancing its bioavailability. Herein, it was noticed that the nanoconjugate (ZnO-PBA-Chry) induced cytotoxicity via combinatorial effect of ZnO-PBA and free chrysin in A549 cells. Therefore, our study indicates that the development of ZnO-PBA-Chry can be an effective agent to inhibit A549 cell survival and proliferation.

## Funding

This research did not receive any specific grant from funding agencies in the public, commercial, or not-for-profit sectors.

## CRediT authorship contribution statement

**Sushweta Mahalanobish**: Conceptualization, Methodology, Software, Data curation, Writing – original draft, Visualization, Investigation, Validation, Writing – review & editing, Formal analysis, **Mousumi Kundu**: Conceptualization, Methodology, Software, Data curation, Writing – original draft, Visualization, Investigation, Validation, Writing – review & editing, Formal analysis, **Sumit Ghosh**: Visualization, Investigation, Writing – review & editing, **Joydeep Das**: Visualization, Investigation, **Parames C. Sil**: Conceptualization, Methodology, Software, Data curation, Supervision, Writing – review & editing, Funding acquisition, Project administration, Resources.

## Declaration of Competing Interest

The authors declare that they have no known competing financial interests or personal relationships that could have appeared to influence the work reported in this paper.

## References

[1] Sompar N., Kukongviriyapan V., Kukongviriyapan U., Senggunprai L., Prawan A. (2015). Protective effects of tetrahydrocurcumin and curcumin against doxorubicin and cadmium-induced cytotoxicity in Chang liver cells. Trop. J. Pharm. Res..

[2] Chowdhury S., Sinha K., Banerjee S., Sil P.C. (2016). Taurine protects cisplatin induced cardiotoxicity by modulating inflammatory and endoplasmic reticulum stress responses. Biofactors.

[3] Mahalanobish S., Saha S., Dutta S., Sil P.C. (2019). Mangiferin alleviates arsenic induced oxidative lung injury via upregulation of the Nrf2-HO1 axis. Food Chem. Toxicol..

[4] Ozben T. (2006). Mechanisms and strategies to overcome multiple drug resistance in cancer. FEBS Lett..

[5] Howard G., Eiges R., Gaudet F., Jaenisch R., Eden A. (2008). Activation and transposition of endogenous retroviral elements in hypomethylation induced tumors in mice. Oncogene.

[6] Grant C.E., Gao M., DeGorter M.K., Cole S.P., Deeley R.G. (2008). Structural determinants of substrate specificity differences between human multidrug resistance protein (MRP) 1 (ABCC1) and MRP3 (ABCC3). Drug Metab. Dispos..

[7] Kim M., Turnquist H., Jackson J., Sgagias M., Yan Y., Gong M., Dean M., Sharp J.G., Cowan K. (2002). The multidrug resistance transporter ABCG2 (breast cancer resistance protein 1) effluxes Hoechst 33342 and is overexpressed in hematopoietic stem cells. Clin. Cancer Res..

[8] Mahalanobish S., Dutta S., Saha S., Sil P.C. (2020). Melatonin induced suppression of ER stress and mitochondrial dysfunction inhibited NLRP3 inflammasome activation in COPD mice. Food Chem. Toxicol..

[9] Ramos S. (2008). Cancer chemoprevention and chemotherapy: dietary polyphenols and signalling pathways. Mol. Nutr. Food Res..

[10] Brannon-Peppas L., Blanchette J.O. (2004). Nanoparticle and targeted systems for cancer therapy. Adv. Drug Deliv. Rev..

[11] Raffa D., Maggio B., Raimondi M.V., Plescia F., Daidone G. (2017). Recent discoveries of anticancer flavonoids. Eur. J. Med. Chem..

[12] Dutta S., Mahalanobish S., Saha S., Ghosh S., Sil P.C. (2019). Natural products: an upcoming therapeutic approach to cancer. Food Chem. Toxicol..

[13] Mohos V., Fliszár-Nyúl E., Ungvári O., Bakos É., Kuffa K., Bencsik T., Zsidó B.Z., Hetényi C., Telbisz Á., Özvegy-Laczka C., Poór M. (2020). Effects of chrysin and its major conjugated metabolites chrysin-7-sulfate and chrysin-7-glucuronide on cytochrome P450 enzymes and on OATP, P-gp, BCRP, and MRP2 transporters, drug metabolism and disposition: the biological fate of chemicals. Drug Metab. Dispos..

[14] Niu B., Liao K., Zhou Y., Wen T., Quan G., Pan X., Wu C. (2021). Application of glutathione depletion in cancer therapy: enhanced ROS-based therapy, ferroptosis, and chemotherapy. Biomaterials.

[15] Walle T., Otake Y., Brubaker J., Walle U., Halushka P. (2001). Disposition and metabolism of the flavonoid chrysin in normal volunteers. Br. J. Clin. Pharmacol..

[16] Brechbuhl H.M., Kachadourian R., Min E., Chan D., Day B.J. (2012). Chrysin enhances doxorubicin-induced cytotoxicity in human lung epithelial cancer cell lines: the role of glutathione. Toxicol. Appl. Pharmacol..

[17] Wang J., Gao S., Wang S., Xu Z., Wei L. (2018). Zinc oxide nanoparticles induce toxicity in CAL 27 oral cancer cell lines by activating PINK1/Parkin-mediated mitophagy. Int. J. Nanomed..

[18] Ghaffari S.-B., Sarrafzadeh M.-H., Fakhroueian Z., Shahriari S., Khorramizadeh M.R. (2017). Functionalization of ZnO nanoparticles by 3-mercaptopropionic acid for aqueous curcumin delivery: synthesis, characterization, and anticancer assessment. Mater. Sci. Eng. C.

[19] Cai X., Luo Y., Yan H., Du D., Lin Y. (2017). pH-responsive ZnO nanocluster for lung cancer chemotherapy. ACS Appl. Mater. Interfaces.

[20] Gong Y., Ji Y., Liu F., Li J., Cao Y. (2017). Cytotoxicity, oxidative stress and inflammation induced by ZnO nanoparticles in endothelial cells: interaction with palmitate or lipopolysaccharide. J. Appl. Toxicol..

[21] Maeda H., Wu J., Sawa T., Matsumura Y., Hori K. (2000). Tumor vascular permeability and the EPR effect in macromolecular therapeutics: a review. J. Control. Release.

[22] Matsumura Y., Maeda H. (1986). A new concept for macromolecular therapeutics in cancer chemotherapy: mechanism of tumoritropic accumulation of proteins and the antitumor agent smancs. Cancer Res..

[23] Wang X., Wei B., Cheng X., Wang J., Tang R. (2016). Phenylboronic acid-decorated gelatin nanoparticles for enhanced tumor targeting and penetration. Nanotechnology.

[24] Geninatti Crich S., Alberti D., Szabo I., Aime S., Djanashvili K. (2013). MRI visualization of melanoma cells by targeting overexpressed sialic acid with a GdIII‐dota‐en‐pba imaging reporter. Angew. Chem..

[25] Liu A., Peng S., Soo J.C., Kuang M., Chen P., Duan H. (2011). Quantum dots with phenylboronic acid tags for specific labeling of sialic acids on living cells. Anal. Chem..

[26] Otsuka H., Uchimura E., Koshino H., Okano T., Kataoka K. (2003). Anomalous binding profile of phenylboronic acid with N-acetylneuraminic acid (Neu5Ac) in aqueous solution with varying pH. J. Am. Chem. Soc..

[27] Muhammad F., Guo M., Qi W., Sun F., Wang A., Guo Y., Zhu G. (2011). pH-Triggered controlled drug release from mesoporous silica nanoparticles via intracelluar dissolution of ZnO nanolids. J. Am. Chem. Soc..

[28] Cai X., Luo Y., Zhang W., Du D., Lin Y. (2016). pH-sensitive ZnO quantum dots-doxorubicin nanoparticles for lung cancer targeted drug delivery. ACS Appl. Mater. Interfaces.

[29] Kundu M., Sadhukhan P., Ghosh N., Chatterjee S., Manna P., Das J., Sil P.C. (2019). pH-responsive and targeted delivery of curcumin via phenylboronic acid-functionalized ZnO nanoparticles for breast cancer therapy. J. Adv. Res..

[30] Kundu M., Chatterjee S., Ghosh N., Manna P., Das J., Sil P.C. (2020). Tumor targeted delivery of umbelliferone via a smart mesoporous silica nanoparticles controlled-release drug delivery system for increased anticancer efficiency, Materials science & engineering. Mater. Sci. Eng. C. Mater. Biol. Appl..

[31] Rodriguez L.G., Wu X., Guan J.-L., Guan J.-L. (2005). Cell Migration: Developmental Methods and Protocols.

[32] Cummings B.S., Wills L.P., Schnellmann R.G. (2012). Measurement of cell death in mammalian cells. Curr. Protoc. Pharmacol..

[33] Sadhukhan P., Kundu M., Chatterjee S., Ghosh N., Manna P., Das J., Sil P.C. (2019). Targeted delivery of quercetin via pH-responsive zinc oxide nanoparticles for breast cancer therapy. Mater. Sci. Eng. C. Mater. Biol. Appl..

[34] Sinha A., Chakraborty A., Jana N.R. (2014). Dextran-gated, multifunctional mesoporous nanoparticle for glucose-responsive and targeted drug delivery. ACS Appl. Mater. Interfaces.

[35] Jorio A. (2012). Raman spectroscopy in graphene-based systems: prototypes for nanoscience and nanometrology. Int. Sch. Res. Not..

[36] Lee S.K., Han M.S., Asokan S., Tung C.H. (2011). Effective gene silencing by multilayered siRNA‐coated gold nanoparticles. Small.

[37] Cui H., Lin Y., Yue L., Zhao X., Liu J. (2011). Differential expression of the α2, 3-sialic acid residues in breast cancer is associated with metastatic potential. Oncol. Rep..

[38] Hsu C.-C., Lin T.-W., Chang W.-W., Wu C.-Y., Lo W.-H., Wang P.-H., Tsai Y.-C. (2005). Soyasaponin-I-modified invasive behavior of cancer by changing cell surface sialic acids. Gynecol. Oncol..

[39] Deshayes S., Cabral H., Ishii T., Miura Y., Kobayashi S., Yamashita T., Matsumoto A., Miyahara Y., Nishiyama N., Kataoka K. (2013). Phenylboronic acid-installed polymeric micelles for targeting sialylated epitopes in solid tumors. J. Am. Chem. Soc..

[40] Sak K. (2014). Cytotoxicity of dietary flavonoids on different human cancer types. Pharmacogn. Rev..

[41] Saha S., Sadhukhan P., Sinha K., Agarwal N., Sil P.C. (2016). Mangiferin attenuates oxidative stress induced renal cell damage through activation of PI3K induced Akt and Nrf-2 mediated signaling pathways. Biochem. Biophys. Rep..

[42] Ma R., Shi L. (2014). Phenylboronic acid-based glucose-responsive polymeric nanoparticles: synthesis and applications in drug delivery. Polym. Chem..

[43] Lee E.Y., Muller W.J. (2010). Oncogenes and tumor suppressor genes. Cold Spring Harb. Perspect. Biol..

[44] Gutschner T., Diederichs S. (2012). The hallmarks of cancer: a long non-coding RNA point of view. RNA Biol..

[45] Marchenko N.D., Moll U.M. (2014). Mitochondrial death functions of p53. Mol. Cell. Oncol..

[46] Wang D.B., Kinoshita C., Kinoshita Y., Morrison R.S. (2014). p53 and mitochondrial function in neurons. Biochim. Biophys. Acta.

[47] Fridman J.S., Lowe S.W. (2003). Control of apoptosis by p53. Oncogene.

[48] Mahalanobish S., Saha S., Dutta S., Sil P.C. (2020). Matrix metalloproteinase: an upcoming therapeutic approach for idiopathic pulmonary fibrosis. Pharmacol. Res..

[49] Fan Y.L., Zheng M., Tang Y.L., Liang X.H. (2013). A new perspective of vasculogenic mimicry: EMT and cancer stem cells. Oncol. Lett..

[50] Liu Q., Qiao L., Liang N., Xie J., Zhang J., Deng G., Luo H., Zhang J. (2016). The relationship between vasculogenic mimicry and epithelial‐mesenchymal transitions. J. Cell. Mol. Med..

